# Primary Gallbladder Lymphoma in a Male Patient with No Risk Factors Detected Incidentally by CT Colonography

**DOI:** 10.1155/2015/813708

**Published:** 2015-10-26

**Authors:** Monil Karia, Grigorios Mitsopoulos, Ketan Patel, Akkib Rafique, Hemant Sheth

**Affiliations:** ^1^Department of Surgery, Ealing Hospital, London UB1 3HW, UK; ^2^Department of Pathology, Ealing Hospital, London UB1 3HW, UK; ^3^Department of Haematology, Hillingdon Hospital, London UB8 3NN, UK; ^4^Department of Radiology, Ealing Hospital, London UB1 3HW, UK

## Abstract

Primary gallbladder lymphoma, although rare, usually presents in females with symptoms mimicking cholecystitis. We present a rare case of primary gallbladder in an 81-year-old male with no risk factors whose only symptom was weight loss. Routine blood tests including liver function tests were unremarkable. A CT colonography was carried out to exclude colonic malignancy. Unilateral gallbladder wall thickening and lymphadenopathy were incidentally detected and confirmed by ultrasound and a decision for the patient to undergo laparoscopic cholecystectomy and intraoperative cholangiogram was made. Histology confirmed extranodal marginal zone lymphoma with follow-up staging and biopsy of the bone marrow not demonstrating spread. Cholecystectomy was therefore deemed curative and no adjuvant therapy was necessary. Thickening of the gallbladder wall on any imaging with or without symptoms should not be ignored or assumed to be cholecystitis, even in males with no risk factors. In these patients urgent cholecystectomy with intraoperative cholangiogram is indicated with histology and haematology follow-up.

## 1. Introduction

Malignant lymphomas are considered tumours of lymph nodes although 40% occur in extranodal tissues, usually from the gastrointestinal tract. As an organ normally devoid of lymphoid tissue primary lymphoma of the gallbladder is exceptionally rare. Usually gallbladder lymphoma is secondary to infiltration from widespread lymphoproliferative disease.

Primary gallbladder cancer is of adenocarcinoma type in 96% of cases with primary lymphoma accounting for only 0.1%–0.2% of cases, the majority of which are diffuse large B cell lymphomas or extranodal marginal zone lymphomas (EMZL) [1]. Most of the literature regarding primary gallbladder lymphoma therefore consists of case reports demonstrating a female preponderance and a presentation mimicking cholecystitis commonly with evidence of gallstones.

In this report we present a rare case of a primary gallbladder lymphoma in a male patient whose only symptom was weight loss and present the incidental radiological findings correlated with the histology and intraoperative findings.

## 2. Case Report

**Figure 1:**
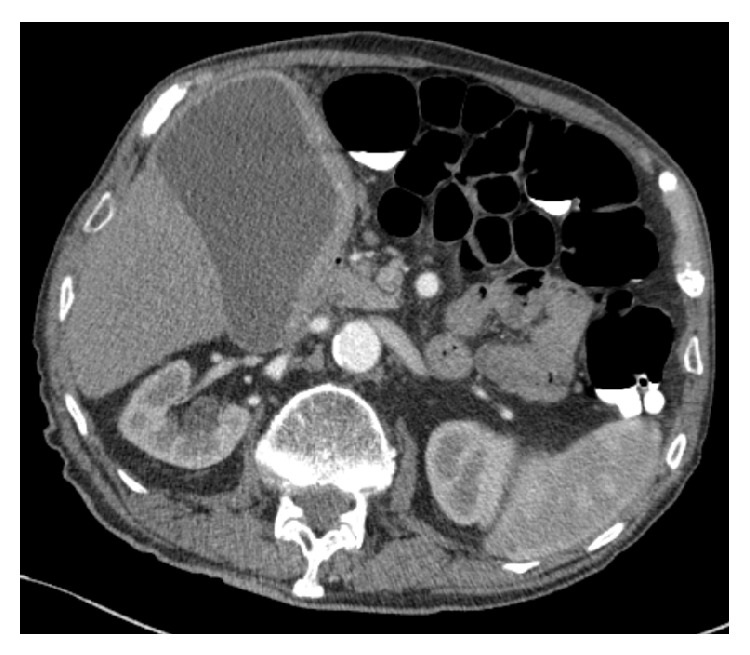
Computerised tomography colonography demonstrating a grossly distended gallbladder with a thickened medial wall measuring up to 1 cm.

An 81-year-old gentleman with early stage Alzheimer's dementia who is otherwise well presented with gradual ongoing weight loss as his only symptom. The patient's full blood count and liver function tests were unremarkable. He underwent virtual CT colonography to investigate the weight loss. Whilst no colonic pathology was found, the imaging detected a distended gallbladder with a thickened medial wall of 1 cm and small volume porta hepatis lymphadenopathy (Figure 1).

**Figure 2:**
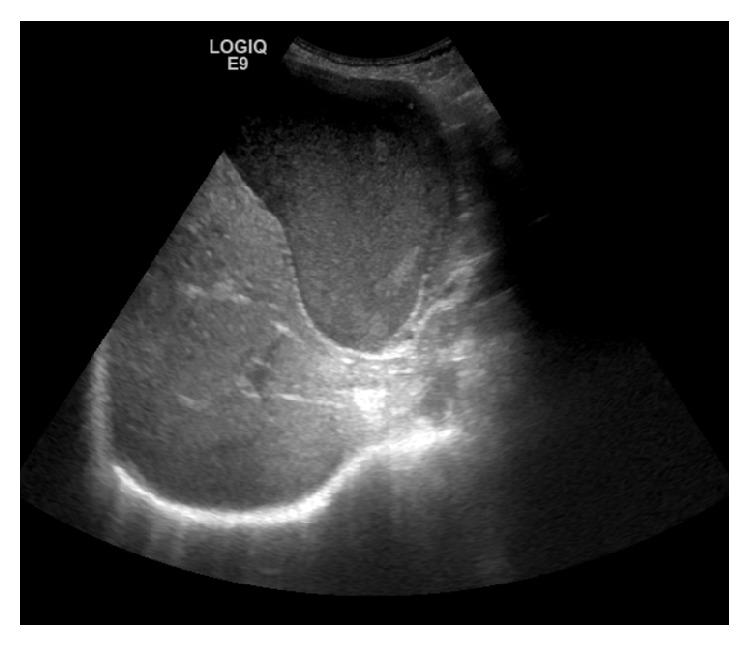
Ultrasound abdomen demonstrating a grossly distended gallbladder with a thickened posterior wall.

This raised the suspicion of gallbladder cancer reaffirmed by an ultrasound scan which demonstrated the gallbladder to be markedly distended containing sludge, with a thickened posterior wall of 8 mm but no gallstones (Figure 2).

The decision was made to proceed to urgent laparoscopic cholecystectomy with on-table cholangiogram. Intraoperative laparoscopy revealed a grossly enlarged, thick-walled gallbladder. To aid dissection percutaneous aspiration was carried out draining over 250 ml of pus. Extensive adhesiolysis to free the gallbladder from the duodenum and colon was required prior to identification of Calot's triangle. An intraoperative cholangiogram was performed demonstrating normal ductal anatomy with no obvious obstruction. The cystic duct and artery were then ligated. Dissection of the gallbladder off the liver bed was exceptionally difficult as it was firmly attached, with complete obliteration of the plane. Once the gallbladder was freed, diathermy to the liver bed was carried out in case any posterior wall remained adherent. Thorough washout was performed and a large drain placed on the liver bed before removing the gallbladder via the umbilical port in a bag. Postoperative recovery was uncomplicated and the patient was discharged with analgesia 72 hours later following drain removal.

**Figure 3:**
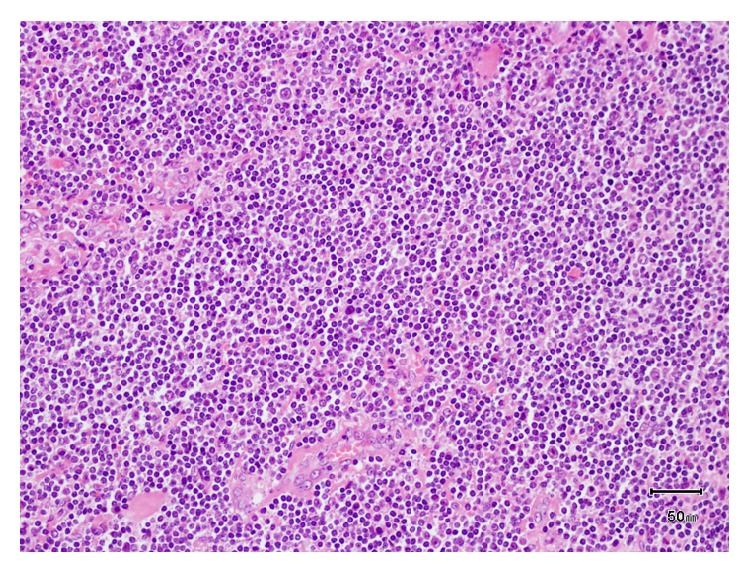
Histology demonstrating lymphoid infiltrate with a diffuse and vaguely nodular growth pattern composed of small, cleaved lymphocytes with centrocyte-like morphology and inconspicuous nucleoli.

Urgent histology revealed replacement of the entire gallbladder by a lymphoid lesion with ulceration of the mucosal surface and transmural infiltration with extension beyond the serosal surface into surrounding adipose tissue. Lymphoid cells were positive for CD 20 and BCL 2 immunostains and negative for CD 3, CD 5, CD 23, CD 10, BCL 6, and cyclin D1 immunostains. CD 21 highlighted expanded follicular dendritic cell MeshWorks. Lymphoid cells showed expression of IgM and were negative for IgD. Overall appearances were those of an extranodal marginal zone lymphoma of the gallbladder (Figure 3).

The patient was referred promptly to haematology, where staging via contrast CT of the neck, chest, abdomen, and pelvis as well as bone marrow studies showed no metastatic disease. The laparoscopic cholecystectomy was considered curative with only routine surveillance required.

## 3. Discussion

Whilst the pathogenesis of EMZL is unclear, various theories have arisen. Tomori et al. studied bile bacteriology and reported that EMZL may develop in 11% of gallbladders in association with positive bile cultures and gallstones [2]. Mani et al. reviewed a small case series and concurred that most gallbladder lymphomas coincide with gallstones and present with cholecystitis secondary to chronic inflammation with cholelithiasis present in over 90% of cases of primary gallbladder lymphoma [3]. This chronic inflammation acting as a “substrate” for primary gallbladder lymphoma causes lymphocytes to migrate to the gallbladder mucosa forming secondary follicles [4]. The continuous antigenic stimulus may cause a chromosomal translocation resulting in a fusion protein that inhibits apoptosis and causes antigen-independent proliferation [5]. Furthermore Mani et al. reported 11/13 prior cases of EMZL occurring in women [3]. This likely reflects the higher prevalence of gallstones in females, which is two to three times higher than in men, attributable to oestrogen increasing biliary cholesterol secretion [6].

In this case our male patient had no evidence of gallstones and lacked any symptoms resembling cholecystitis. Gallbladder lymphoma should not, therefore, be excluded if the patient lacks these findings, since presentations of this condition can vary. Our patient presented indolently with weight loss and the diagnosis was picked up incidentally. Similarly a recent report demonstrated a gallbladder lymphoma which grew to 4 cm with relatively little clinical signs and normal serum levels of carcinoembryonic antigen (CEA) and carbohydrate antigen (CA) 19-9 [7]. Despite the lack of clinical signs, intraoperatively our patient's gallbladder was distended and filled with pus. Friedman et al. also presented a case of gallbladder lymphoma with an empyema but in this case associated with a lung abscess on the same side resulting in severe sepsis which the patient did not survive [8].

As with gallbladder carcinoma, ultrasonography is sufficient for identification of wall thickening, polyps, or masses prior to surgery. More specifically, CT or magnetic resonance imaging (MRI) findings of homogeneous submucosal thickening of the gallbladder wall with a preserved mucosal surface are described as being suggestive of lymphoma [9]. Fine needle aspiration has also been presented as a potential preoperative diagnostic tool [10]; however proceeding with urgent cholecystectomy for histological diagnosis is still best practice. Postoperative staging with whole body CT and bone marrow aspiration/trephine biopsy should be completed as per lymphoma work-up. If the cancer is confined to the gallbladder, then, whilst adjuvant irradiation and chemotherapy have been reported to have good effect, more recent evidence from the literature is now largely suggesting that cholecystectomy alone is sufficient for long term disease-free survival [11]. These tumours therefore have an excellent prognosis with complete resection. We also strongly recommend intraoperative cholangiogram as lymphomas of the extrahepatic ducts have been reported [12].

Primary extranodal marginal zone lymphoma of the gallbladder, whilst rare, has an excellent prognosis if detected early and resected via laparoscopic cholecystectomy. Whilst presentation is most commonly in females with gallstones and symptoms mimicking cholecystitis we present a case in a male with no such findings. Thickening of the gallbladder wall on any imaging with or without symptoms should not be ignored or assumed to be cholecystitis, particularly in the elderly, even when no risk factors are present. With these findings, patients should instead undergo urgent cholecystectomy, with or without stones and even with normal blood results. Moreover, intraoperative cholangiogram is beneficial in excluding bile duct lymphoma and urgent histology should always be requested so that postoperative referral to haematology can be expedited.
